# Bronchopneumonia in Swedish lambs: a study of pathological changes and bacteriological agents

**DOI:** 10.1186/s13028-018-0409-1

**Published:** 2018-09-17

**Authors:** Lisa Lindström, Felicia Asp Tauni, Karin Vargmar

**Affiliations:** Department of Biomedical Sciences and Veterinary Public Health, Faculty of Veterinary Medicine and Animal Science, Swedish University of Agricultural Sciences (Ulls väg 26), 757 56 Uppsala, Sweden

**Keywords:** Chronic bronchopneumonia, Atypical pneumonia, Lamb, *Mycoplasma ovipneumoniae*, Respiratory disease

## Abstract

**Background:**

One of the most common post-mortem inspection finding of sheep and lambs in Sweden, following routine slaughter is pneumonia and its prevalence is increasing. To our knowledge, the aetiology of pneumonia in lambs is not well-known for Swedish conditions. Chronic bronchopneumonia, also known as “atypical” or chronic non-progressive pneumonia, is a common disease worldwide, affecting lambs up to 12 months old. It is therefore of interest to elucidate if this disease complex is also a common cause of pneumonia among Swedish lambs. Chronic bronchopneumonia has a characteristic macroscopic and histopathologic appearance, and *Mycoplasma ovipneumoniae* is the microbial agent most frequently found. Although this bacterium is important for the pathogenesis, multiple agents are presumed to be involved. The aim of this study was to describe the macroscopic and histopathologic lung lesions in routinely slaughtered lambs with pneumonia, and to determine the bacterial agents involved.

**Results:**

A total of 41 lungs with gross lesions consistent with pneumonia were examined. Of these, 35 lungs displayed the typical gross appearance of chronic bronchopneumonia, with several or all of the characteristic histological features. *M. ovipneumoniae* was detected in 83% of the 35 lungs and *Mannheimia haemolytica* was isolated in 71%. Pneumonia associated with *M. ovipneumoniae* could be correlated to specific gross lesions consistent with the gross description of chronic bronchopneumonia in lambs.

**Conclusion:**

In this study, chronic bronchopneumonia was the most common lung disease in routinely slaughtered Swedish lambs. This diagnosis was based on the characteristic macroscopic and histopathologic pulmonary findings and the frequent presence of the bacterium *M. ovipneumoniae*. The macroscopic appearance of chronic bronchopneumonia could therefore be used during routine investigation of the lamb carcasses at slaughter, to determine the most likely cause of pneumonia.

## Background

Swedish lamb production is growing, with both the number of animals and farmers steadily increasing. Although Swedish sheep and lambs are generally healthy, pneumonia and pleuritis are two of the most common findings during routine slaughter of lambs. The prevalence of these illnesses increased from 1.2% in 2005 to 2.8% in 2015 [1]. Between 2011 and 2013 Gård and Djurhälsan (Farm and Animal Health) financed 2200 necropsies of sheep and lambs. They showed that 20% of the sheep and 15% of the lambs (2–12 months old) suffered from pneumonia [2]. However, since the data were collected from animals sent for necropsy, it could be assumed that they had presented with severe signs of disease, had a therapeutic failure, or had been found dead. To our knowledge, the aetiology causing pneumonia in lambs is not well-known for Swedish conditions.

In lambs that are up to 12 months old, chronic bronchopneumonia (also called atypical pneumonia, chronic non-progressive bronchopneumonia, and mycoplasmosis), is a common, often subclinical disease, worldwide. Multiple infectious agents are implicated, including *Mycoplasma ovipneumoniae*, *Mannheimia haemolytica*, and possible viral agents, like parainfluenza type 3 virus, and respiratory syncytial virus [3–5]. *M. ovipneumoniae* is the agent most frequently found [6–8]. Thus, it can be assumed to play an important role in the pathogenesis of atypical pneumonia. *M. ovipneumoniae* is mainly transmitted via the respiratory tract through close contact between the animals [6], and lambs are infected within a few days of birth [4]. The bacteria can be isolated in the respiratory tract of healthy animals, suggesting it is part of the normal bacterial flora [9, 10]. However, primary infection with the bacteria might predispose invasion by other organisms, such as *M. haemolytica* [6, 10]. *M. ovipneumoniae* can predispose to secondary infection by producing hydrogen peroxide, causing ciliostasis [11] and facilitating the mycoplasma infection. The pathogenesis of *M. ovipneumoniae* involves a polysaccharide capsule, which could promote adherence of the organisms to the ciliated epithelium, thus promoting bacterial colonization [12, 13]. Furthermore, the polysaccharide capsule may have cytotoxic effects on the epithelial cells, inducing apoptosis [14, 15], which would also contribute to the pathogenesis of the disease. *Mycoplasma* spp. lack cell walls, making the bacteria resistant to penicillin, so treatment would require broad-spectrum antibiotics [16, 17]. Because of the risk of antibiotic resistance, unnecessary use of antibiotics should be avoided. Stress, poor air quality and adverse weather conditions may also contribute to the development of the disease [3, 6]. Apart from the effect on the individual animal’s health, chronic bronchopneumonia has economic impacts due to reduced growth rates in the lambs [18–20].

Chronic bronchopneumonia causes lesions in the cranial lung lobes. These can be observed as red-brown discolorations with clearly demarcated regions of consolidation and occasional pleuritis [6, 21, 22]. The typical finding in histopathological examinations is a chronic active inflammation. It is characterized by lymphoid proliferation around airways, bronchiolar epithelial hyperplasia, neutrophil accumulation in airspaces, and the unique lesion of hyaline scars. The hyaline scars consist of nodular masses of collagen and fibroblasts in the wall of bronchioles that protrude into the lumen [3, 22, 23].

In Sweden, all lungs from sheep and lambs rejected at slaughter are coded with one of three specific codes representing the cause of pneumonia. These are: lesion from lungworm (code 73/74), pleuritis and pericarditis (code 75/76) and “other pneumonia” (code 63/64). Previously there was also a code for chronic bronchopneumonia or “mycoplasma-like pneumonia” (code 61/62). It was assigned to lungs with a gross appearance of moderate pneumonia in at least three lung lobes, or severe pneumonia in one lung lobe. However, this code is no longer in use.

Studies from other countries have shown that chronic bronchopneumonia is common in slaughtered lambs [6, 7, 24]. Therefore, it is of interest to investigate if the disease complex is also common in the Swedish lamb population. The aim of this study was to investigate the bacterial agents found in lungs with pneumonia detected at routine slaughter of lambs, and to describe the macroscopic and histopathological appearance of the lesions. The study also aimed to elucidate if the macroscopic appearance of chronic bronchopneumonia could establish the cause of disease when applied to Swedish flocks. If it can be determined during routine investigations of carcasses at slaughter the farmer would gain valuable information about flock health and it would contribute to national disease surveillance.

## Methods

### Sample collection

During the period of September–October 2016, all lamb lungs with pneumonic lesions at post-mortem inspection were collected from two slaughterhouses located in the province of Uppland, Sweden. In total, 44 sets of lungs with lesions were collected. Of these, 41 had gross lesions suggestive of pneumonia and were included in the study. The lungs were stored at maximum + 4 °C until starting the examination and sampling, which were performed within 2 days of slaughtering. The lambs in the study passed the ante-mortem inspection performed by a veterinarian. Therefore, it was assumed that the animals had shown few or no clinical signs of disease prior to slaughter. No details of the sex, breed or husbandry conditions were available.

### Pathological investigation

**Fig. 1:**
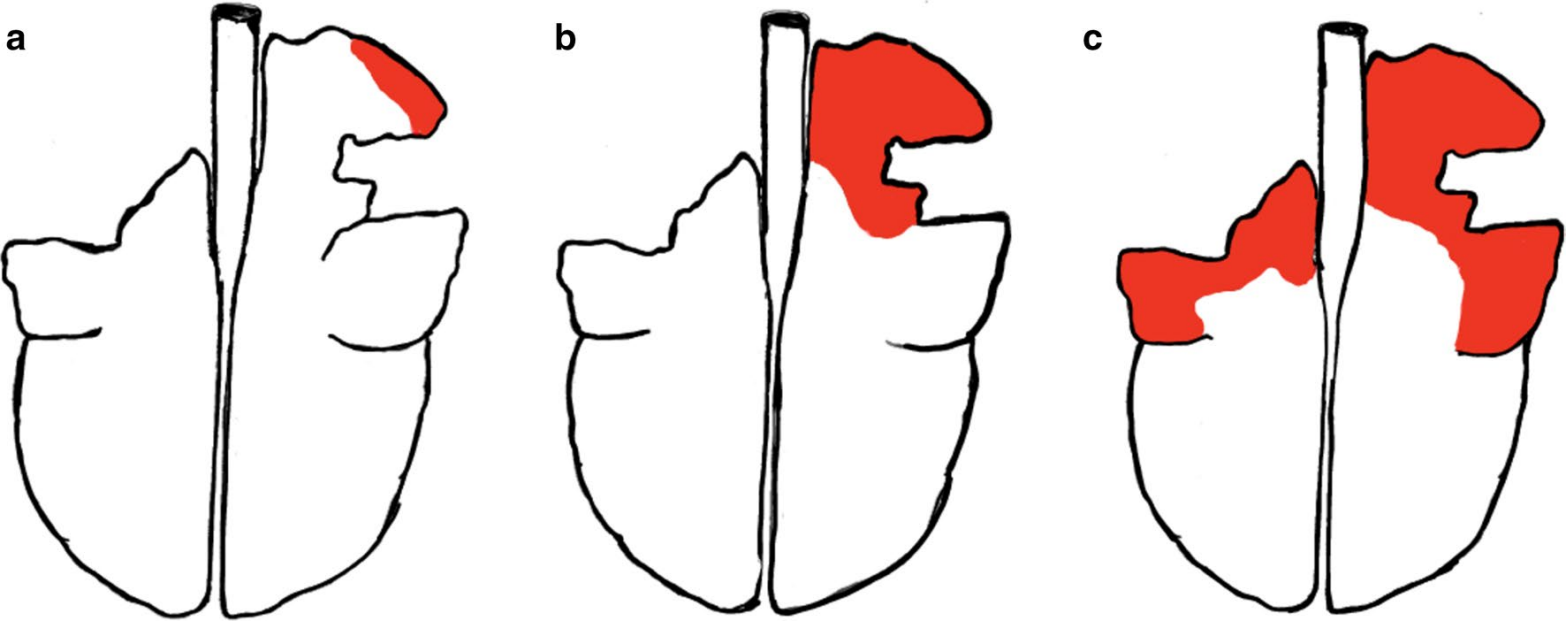
Examples of the grading system of the pneumonia. **a** Less the 10% of the tissue affected, pneumonia scored as mild. **b** 10–20% of the tissue affected, pneumonia scored as moderate. **c** More than 20% of the tissue affected, pneumonia scored as severe

The pulmonary lesions were photographed and described using the following attributes: distribution, demarcation, contour, shape, colour, size, texture, consistency, and extent. A schematic illustration was used to get an objective estimate of the extent of consolidation (Fig. 1). The affected areas were marked with red, and the graphics editor GNU Image Manipulation Program (GIMP 2.10.2) was used to calculate the areas of the red markings. The pneumonia was then scored as mild, moderate, or severe, depending on the extent of the consolidation. If < 10% was affected the pneumonia was scored as mild, 10–20% as moderate and > 20% as severe. The same categories were used by Sheehan et al. [24]. Type 1 lungs were classified as having at least the right cranial lung lobe exhibiting the following attributes: well-demarcated homogeneous consolidation, red-brown discolorations, and meaty consistency. These lesions are characteristic of chronic bronchopneumonia [6, 22]. The lungs with lesions that did not fit the description were grouped as Type 2. Representative samples of grossly affected lung tissue were fixed in 10% buffered formalin.

The formalin fixed tissue was embedded in paraffin and cut in 4-μm sections. These were stained with haematoxylin and eosin and examined with light microscopy. If fibrosis was suspected, the slides were stained with Masson’s trichrome [25]. The histopathological investigations were blinded. Thus, no information about the gross appearance of the lungs or microbiological results were available at the time of histopathological examination. General characteristics of the inflammatory process that were recorded included pattern, chronicity, and extent of the lesion. It was also recorded if there was pleuritis, hyperplasia of the bronchiolar epithelium, hyperplasia of lymphoid tissue, exudate in the lumen of the airways, or hyaline scars.

### Microbiological investigation

Affected lung tissue, (usually the right cranial lobe) was cut, using a sterile scalpel. Thereafter a Liquid-based Collection and Transport System (E-Swab, COPAN Diagnostics) was used to collect samples from the lung parenchyma at the cut surface. One E-swab was used for bacterial isolation and one for detection of mycoplasma by polymerase chain reaction (PCR).

The bacterial isolations were performed at the diagnostic laboratory at SVA (the Swedish National Veterinary Institute) using accredited methods including bacterial culture for MALDI-TOF MS (Matrix Assisted Laser Desorption Ionization Time-of-Flight Mass Spectrometry) (Bruker Daltonic GmbH, Bremen, Germany). The analyses started within 24 h of sample collection. The laboratory was requested to pay particular attention to any growth of *Pasteurella multocida, M. haemolytica, and Bibersteinia trehalosi* and an antimicrobial susceptibility test was performed on collected bacterial agents. The same breakpoints for resistance were used as in the report from the Swedish Veterinary Antimicrobial Resistance Monitoring [26]. One of the samples taken from the cut surface using the Liquid-based Collection and Transport System (E-Swab) was sent to the Animal and Plant Health Agency (Department of Bacteriology, Mycoplasma Group, Weybridge, UK) where the PCR detection of *Mycoplasma* spp. was performed [27].

### Statistics

The statistical analyses were done at SLU (Swedish University of Agricultural Sciences) Centre for Statistics using Fischer’s exact test (SAS^®^ System for Windows, version 9.3; SAS Institute Inc., Cary, NC, USA) and the result was interpreted as significant at a P-value of < 0.05.

## Results

### Pathological investigation

#### Gross pathological investigation

During the sampling period, a total of 44 lamb lungs displayed signs of lung pathology and were collected from the slaughterhouses. Three lungs did not show the characteristics of pneumonia and were excluded from the study. The gross appearances for the remaining 41 lungs were described and the extent of the consolidated area was calculated to evaluate the severity. The severity of the pneumonia was scored as mild in 12%, moderate in 73%, and severe in 17% of the lungs. Examples of the grading system are demonstrated in Fig. 1.

**Fig. 2:**
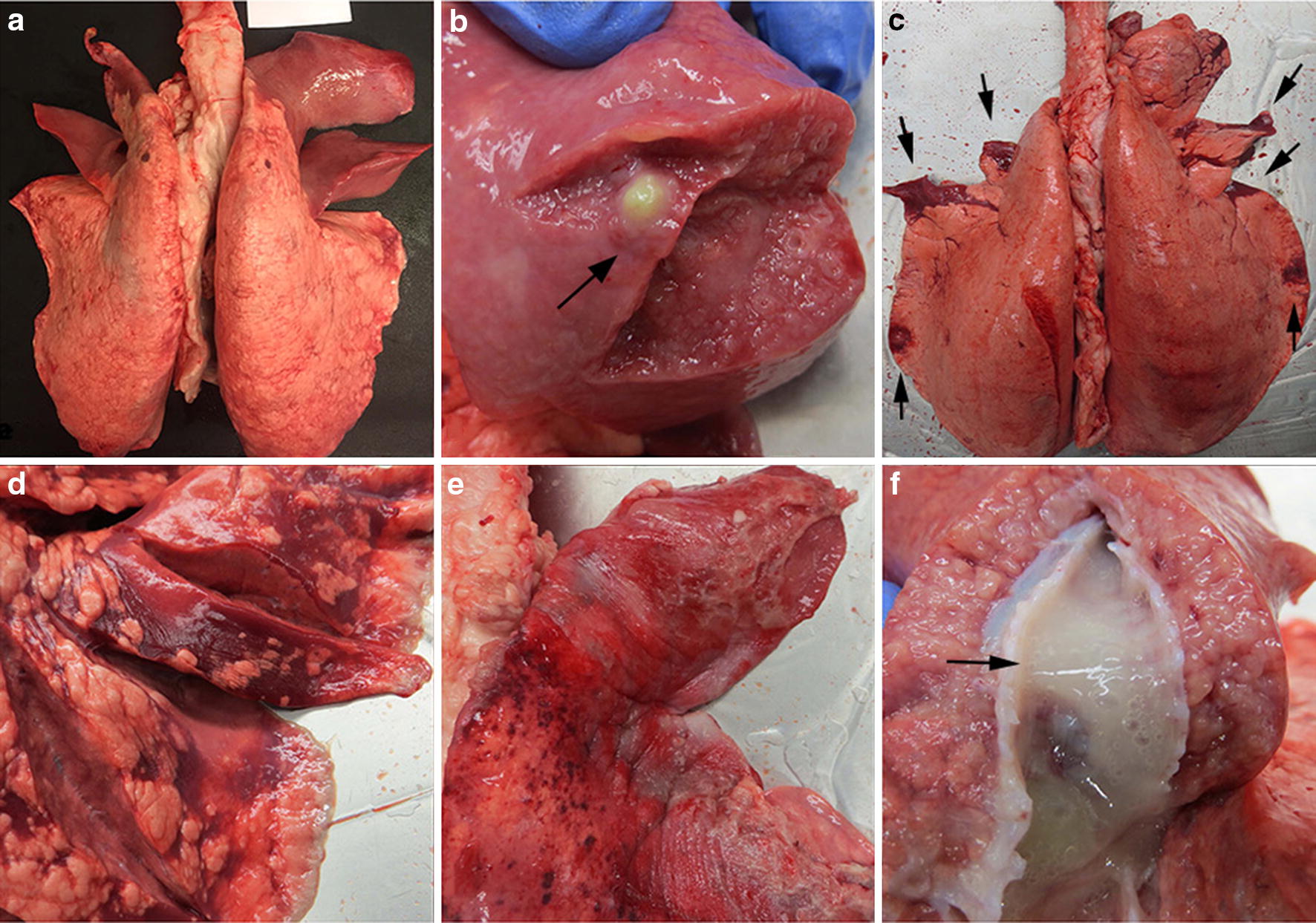
Examples of macroscopic lesions in the lungs. **a** Type 1 lung. Well-demarcated consolidated areas and red-grey discoloration affecting the cranial lung lobes, lung number 1. **b** Type 1 lung. Well-demarcated consolidated areas and red-grey discoloration and abscess (black arrow), lung number 19. **c** Type 2 lung. Multifocal consolidated areas affecting both cranial and caudal lung lobes (black arrows), lung number 6. **d** Type 2 lung. Mainly subpleural consolidation of the tissue, lung number 9. **e** Pleuritis, lung number 8. **f** Mucopurulent exudate within airways (black arrow), lung number 18

Out of the 41 lungs, 35 showed well demarcated consolidated areas with cranial distribution affecting one or several lobules. The lesions were red-brown to purple-grey and had a firm, meaty consistency. The texture was solid, and the cut surfaces varied from dark red and oedematous to greyish and firm, occasionally with pale grey areas (Fig. 2a, b). In five cases multifocal abscesses were observed (Fig. 2b). All 35 lungs fitted the classification criteria of Type 1. The gross appearances of Type 1 lesions were consistent with the characteristics of chronic bronchopneumonia [3, 6, 23]. Out of the 41 lungs, six did not fulfil the Type 1 criteria and were therefore classified as Type 2. For example, the Type 2 lungs had lesions with multifocal distribution affecting both cranial and caudal lung lobes (Fig. 2c), and lesions mainly affecting subpleural areas of the lungs (Fig. 2d). Out of the 41 lungs, 19 (46%) had grossly visible pleuritis (Fig. 2e), and in 25 (61%) clearly visible mucous to mucopurulent exudate was present in the airways (Fig. 2f). The macroscopic appearances are summarised in Table 1.

**Table 1:** Summary of pathological and microbiological findings

No.	Gross pathology					Histopathology					Microorganisms		
	S	T	GPl	GEx	Ab	HPl	EH	LH	HEx	HS	Isolation	PcS	Myc PCR
1	sev	1	−	+	−	−	+	+	+	+	*M.h*	S	*M.o*
2	sev	1	+	+	+	+	+	+	+	+	–		–
3	mod	2	−	+	−	−	+	+	+	+	*S.e*	S	*M.o*, *M.a*
4	mod	1	−	+	−	−	+	+	+	−	*M.h*	S	*M.o*
5	mod	1	−	−	−	−	+	+	+	−	–		–
6	mod	2	−	−	+	−	−	+	−	−	–		–
7	mod	1	−	−	−	+	+	+	+	−	*M.h*	S	*M.o*
8	sev	1	+	+	−	+	+	+	+	+	*M.h*	S	*M.o*
9	mld	2	+	−	−	+	+	+	+	−	–		–
10	mod	1	+	+	−	+	+	+	+	+	*M.h*	S	*M.o*
11	sev	1	−	+	−	+	+	+	+	−	–		*M.o*
12	mod	1	−	−	−	+	+	+	+	−	*M.h*	S	*M.o*
13	mld	1	+	+	−	+	+	−	+	−	–		*M.o*
14	mld	1	+	−	−	+	+	+	+	−	–		*M.o*
15	mod	1	+	+	−	+	+	+	+	−	*M.h*	S	*M.o*
16	mld	2	+	−	−	+	+	+	−	−	–		–
17	mod	1	+	−	−	+	+	+	+	−	*M.h*	S	*M.o*
18	mod	1	−	+	−	+	+	+	+	−	*M.h*	S	*M.o*
19	mod	1	+	+	+	+	+	+	+	−	–		*M.o*
20	mod	2	−	−	−	+	+	+	+	−	–		–
21	sev	1	+	+	−	+	+	+	+	−	–		–
22	mod	1	−	+	−	−	+	+	+	+	*M.h*	S	*M.o*, *M.a*
23	mod	1	−	+	−	+	+	+	+	−	*M.h*	S	*M.o, M.a*
24	mod	1	−	−	−	−	+	+	+	+	–		*M.o*
25	sev	1	−	−	−	−	+	+	+	−	*M.h*	S	*M.o*
26	mld	2	−	+	−	+	+	+	+	−	*M.h*	S	*M.o*
27	mod	1	+	+	−	−	+	+	+	−	*M.h*	S	*M.o*, *M.a*
28	mod	1	−	+	−	−	+	+	+	−	–		*M.o*
29	sev	1	+	−	−	−	+	+	+	+	*M.h*	S	*M.o*
30	mod	1	+	+	+	+	+	+	+	+	*B.t*	S	*M.a*
31	mod	1	+	+	+	+	+	+	+	+	*M.h*	S	*M.a*
32	mod	1	−	+	−	+	+	+	+	+	*M.h*	S	*M.o*
33	mod	1	−	−	−	+	+	+	+	−	*M.h*	S	*M.o*
34	mod	1	+	−	−	+	+	+	+	−	*M.h*	S	*M.a*
35	mod	1	−	+	−	−	+	+	+	+	*M.h*	S	*M.o*
36	mod	1	−	+	−	−	+	+	+	−	*M.h*	S	*M.o*
37	mod	1	−	+	−	+	+	+	+	+	*M.h*	S	*M.o*, *M.a*
38	mod	1	−	−	−	+	+	+	+	−	*M.h*	S	*M.o*
39	mod	1	+	+	−	+	+	+	+	+	*M.h*	S	*M.o*
40	mod	1	+	−	−	+	+	+	+	−	*M.h*	S	*M.o*
41	mod	1	+	+	−	+	+	+	+	−	*M.h*	S	*M.o*

S, severity of lesions; mld, mild; mod, moderate; sev, severe; T, type of appearance; 1, Type 1; 2, Type 2; +, present; −, absent; GPl, evidence of pleuritis at gross examination; GEx, signs of exudate in airways at gross examination; Ab, abscess; HPl, visible pleuritis in histopathological examination; EH, bronchiolar epithelial hyperplasia; LH, peribronchial/peribronchiolar lymphoid hyperplasia; HEx, Exudate whitin airways; HS, hyalin scar; Isolation, isolation of bacteria; PcS, penicillin sensitivity of isolated bacteria (S, sensitive); Myc PCR, Mycoplasma PCR; *M.h*, *M. haemolytica* isolated; *S.e*, *S. equisimilis* isolated; *B.t*, *B. trehalosi* isolated; *M.o*, *M. ovipneumoniae* detected; *M.a*, *M. arginini* detected

#### Histopathological investigation

**Fig. 3:**
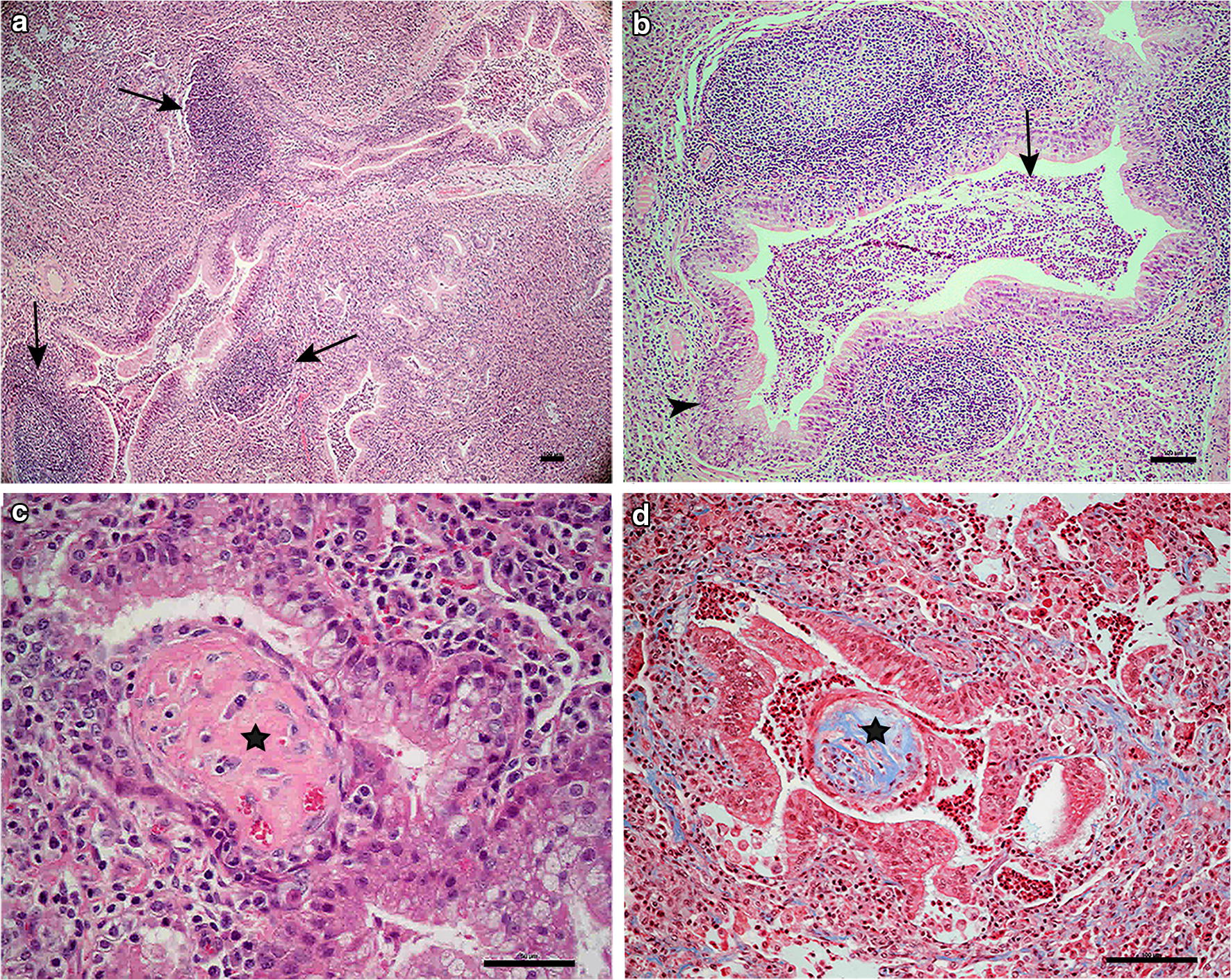
Examples of the histopathological changes in the lungs. **a** Severe consolidation of the tissue and lymphoid proliferation (black arrows) around airways, HE, bar 100 μm. **b** Hyperplasia of the bronchiolar epithelium (black arrowhead) and neutrophilic exudate within airspaces (black arrow) HE, bar 100 μm. **c** Hyaline scar (black star) HE, bar 50 μm. **d** Hyaline scar (black star). Masson Trichrome (connective tissue stains with blue colour), bar 100 μm

All 41 lung slides were dominated by severe consolidation (Fig. 3a). The inflammation was interpreted as either chronic or chronic active. Apart from consolidation, the most common findings were bronchiolar epithelial hyperplasia (98%), peribronchial/peribronchiolar lymphoid hyperplasia (98%), and exudate dominated by neutrophils within the airspaces (95%) (Fig. 3a, b). Hyaline scars were observed in 14 lungs (34%) (Fig. 3c). The scars were most easily detected with the Masson’s trichrome stain (Fig. 3d). Pleuritis was evident in 28 of the lungs (68%), and in most, it was mild and chronic.

Other, less frequently observed lesions, included proliferation of type II pneumocytes and bronchiectasis. An incidental finding was present in one lung; it had remnants of lungworms but with no inflammatory lesions adjacent to the parasite. A summary of the results from the histopathological examination is presented in Table 1.

#### Microbiological investigation

*Mycoplasma ovipneumoniae* was detected in 76% (31/41) and *Mycoplasma arginini* in 20% (8/41) of the lungs*. M. ovipneumoniae* was present in 83% (29/35) of the lungs classified as Type 1. *M. haemolytica* was isolated in 63% (26/41), always together with a *Mycoplasma* spp. (*M. ovipneumoniae* and/or *M. arginini*), most often with *M. ovipneumoniae. Streptococcus dysgalactiae* subsp. *equisimilis* was isolated from one lung.

Lungs number 30 and 31 had multiple abscesses. In these two cases, two samples were taken, one from the cut surface of the consolidated parenchyma and one from the abscess. In lung number 30, *M. arginini* was detected from the parenchyma and *B. trehalosi* was isolated from the abscess. In lung number 31, *M. haemolytica* was isolated and *M. arginini* was detected from the parenchyma. No specific bacterial agent could be isolated from the abscess.

In seven lungs (three Type 1 lungs, and four Type 2 lungs), no bacterial agent was found. The isolates of *M. haemolytica*, *B. trehalosi* and *S. dysgalactiae* found in the affected tissue were all sensitive to penicillin. A summary of the results from the microbiological investigation is presented in Table 1.

There was a statistically significant association between PCR positive samples for *M. ovipneumoniae* and the gross appearance classified as Type 1 (Fischer’s exact test P = 0.0217). There was no significant association between *M. ovipneumoniae* and any of the specific histological attributes. No significant correlations were found between the presence of *M. haemolytica* and any of the gross or histopathological findings.

## Discussion

Out of the collected lungs, 85% were classified as Type 1, macroscopically suggestive of chronic bronchopneumonia. *M. ovipneumoniae* was the most frequent bacterium found (83%), followed by *M. haemolytica* (71%). These bacteria, particularly *M. ovipneumoniae*, are part of the aetiology of chronic bronchopneumonia in lambs [3, 6, 23], further supporting the diagnosis. Previous studies have pointed out that *M. ovipneumoniae* can be found in lungs without gross evidence of pneumonia. However, the prevalence of this bacterium is higher in diseased lungs [6, 7], and with larger amounts of bacteria [6, 22]. In our study, *M. ovipneumoniae* was detected using PCR, which does not provide information about the amount of bacteria in the affected tissue. Due to limited possibility for sampling of healthy lungs as paired controls, our study lacks information concerning prevalence. Nevertheless, *M. ovipneumoniae* was found in a high proportion of affected lungs, suggesting a non-negligible role in the disease complex. Type 1 lungs exhibited all, or several, of the histopathological lesions strongly associated with chronic bronchopneumonia including: lymphoid proliferation, bronchiolar epithelial hyperplasia, hyaline scars, and exudate in airways [22, 23].

Out of the six lungs classified as Type 2, *M. ovipneumoniae* was identified in two lungs, and in one of these in combination with *M. haemolytica.* During microscopic evaluation, all six cases exhibited several of the different histological lesions described in chronic bronchopneumonia. One cannot therefore exclude that those lungs, although lacking the typical gross appearance, represent this disease complex. The deviant gross appearance might represent older stages of a healing process. Furthermore, Type 2 lungs were an exclusion category, and other aetiologies are conceivable.

*Mycoplasma arginini* was present in 20% (8/41) of the lungs examined. In five cases it was found together with *M. ovipneumoniae* and *M. haemolytica*. In two cases it was found solely with *M. haemolytica*. Finally, it was found in the parenchyma of the single lung where *B. trehalosi* was isolated from an abscess. The pathogenicity of *M. arginini* is not completely known, and most often the organism is considered non-pathogenic [10, 28, 29]. The bacterium has been shown to cause elevation in rectal temperature, circulating monocytes, neutrophils and blood fibrinogen [28]. However, the bacterium did not induce any tissue lesion in the same study. In our study, *M. arginini* was observed in all of our cases in the presence of additional bacteria, supporting its role as a non- or minimally pathogenic bacterium.

Seven of the lungs in this study were found to have no bacterial agent. The gross classifications of these lungs were both of Type 1 (three lungs) and Type 2 (four lungs). Possible reasons could be that there were no viable bacteria in the sampled area, the sample handling had been inadequate, or the pneumonia was of viral aetiology. Chronic bronchopneumonia in lambs is described as a multifactorial disease and both parainfluenza virus type 3 and respiratory syncytial virus are suggested to be part of the aetiology. These viruses cause mild respiratory disease with fever, anorexia, and sporadic coughing [30]. Parainfluenza virus type 3 [30, 31] and respiratory syncytial virus are only reported to cause serious diseases in co-infections with additional pathogens [30]. Because of the suggested minor role of viruses, we decided to focus on bacterial agents, as these are believed to be the most important cause of disease. However, the lungs were not tested for viruses, so we cannot exclude that a concurrent viral infection may have contributed to some of the lung lesions.

The pneumonia was evaluated as moderate (73%) or severe (17%) in a high proportion of the lungs, and 95% had neutrophilic exudate within airspaces. This might have been a consequence of the common co-infection with *M. haemolytica. M. ovipneumoniae* causes mild suppuration [32] and in co-infection with *M. haemolytica*, the neutrophilic exudation is enhanced [21, 24]. The high incidence of pneumonic lesions classified as moderate or severe is noteworthy since the lambs passed the ante-mortem inspection and might reflect the difficulty in recognizing sick animals. This further highlights the importance of adequate post-mortem registration of lesions in disease surveillance.

## Conclusions

In this study, chronic bronchopneumonia was the most common lung disease in routinely slaughtered lambs in the province of Uppland, Sweden. In affected lungs, *M. ovipneumoniae* was a common pathogen and often occurred in combination with *M. haemolytica*. In the literature, these bacteria, in particular *M. ovipneumoniae,* are frequently associated with the disease complex [4, 6, 8]. A high proportion of the lungs showed the characteristic macroscopic appearance of chronic bronchopneumonia and the lungs exhibited all or several of the typical histological features described for the disease, further supporting the diagnosis. The macroscopic appearance of chronic bronchopneumonia could therefore be used as an indicator of the most likely cause of pneumonia during post-mortem investigation of the carcasses of Swedish lambs.
